# Microtubule Minus-End Binding Proteins in Cancer: Advances

**DOI:** 10.3390/diagnostics15243116

**Published:** 2025-12-08

**Authors:** Qingwen Wang, Xiuling Li, Meng Xie, Xiangming Ding, Dongxiao Li

**Affiliations:** Department of Gastroenterology, Zhengzhou University People’s Hospital, Henan Provincial People’s Hospital, Zhengzhou 450003, China; qingwen936@163.com (Q.W.); zzlixiuling@aliyun.com (X.L.); xiemeng@zzu.edu.cn (M.X.); dingxiangming@zzu.edu.cn (X.D.)

**Keywords:** microtubule minus-end binding proteins, microtubules, tumor, prognosis

## Abstract

Microtubule minus-end binding proteins (−TIPs) are critical regulators of microtubule dynamics and stability, whose dysfunctions are increasingly associated with tumorigenesis and cancer progression. This review systematically consolidates current research advances on the molecular characteristics, oncogenic mechanisms, and therapeutic potential of −TIPs in cancer. By integrating preclinical studies, multi-omics data, and clinical evidence, it was found that calmodulin-regulated spectrin-associated proteins (CAMSAPs) and abnormal spindle microtubule assembly (ASPM) primarily exhibit oncogenic properties, whereas CAMSAP3 acts as a tumor suppressor by negatively regulating tumor cell migration. Studies also demonstrate that pharmacological inhibition of the γ-tubulin ring complex (γ-TuRC) effectively attenuates the centrosomal hyper-clustering capacity of malignant cells, thereby suppressing invasive phenotypes. This result underscores the therapeutic value of targeting −TIPs. In summary, −TIPs play critical and complex roles in cancer progression and hold significant potential as prognostic biomarkers and therapeutic targets. Intervention strategies focusing on specific −TIPs, such as γ-TuRC, offer promising strategies for precision cancer therapy; however, the context-dependent functions of these proteins require further investigation to facilitate clinical translation.

## 1. Introduction

**Figure 1:**
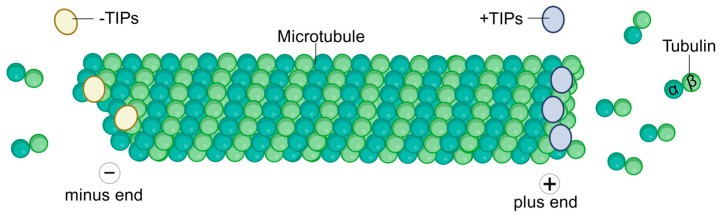
Schematic representation of a microtubule and its end-binding proteins. Microtubules are polarized filaments with a dynamic plus-end and a relatively stable minus-end. +TIP accumulate at the growing plus-end. −TIPs associate with and stabilize the minus-end.

As fundamental components of the eukaryotic cytoskeleton, microtubules constitute polarized tubular polymers assembled through the dynamic association of α/β-tubulin heterodimers. The β-tubulin-exposed terminus (designated as the plus-end) exhibits faster polymerization kinetics, whereas the α-tubulin-exposed terminus (minus-end) demonstrates slower growth dynamics. Through dynamic polymerization/depolymerization cycles, microtubules coordinate essential cellular processes including mitotic progression, intracellular trafficking, signal transduction, and polarity establishment, with precise regulation of their dynamic instability being paramount for cellular homeostasis. Research efforts have predominantly focused on plus-end binding proteins (+TIPs) such as CLIP-170 and EBs, whereas systematic understanding of minus-end regulation remains comparatively limited. Recent advancements in super-resolution imaging and cryo-electron tomography, coupled with genomic profiling approaches, have propelled −TIPs to the forefront of cytoskeletal research [1,2,3,4]. These regulatory proteins orchestrate microtubule nucleation and spatial organization through minus-end anchoring, stabilization, and post-translational modifications, thereby governing mitotic fidelity, directional migration, and polarized growth [2,5,6,7,8,9,10,11]. A schematic diagram of a microtubule, highlighting its polarity and the binding locations of +TIPs and −TIPs, is shown in Figure 1.

Dysregulation of −TIPs has been pathologically linked to multiple disease states, with oncogenic processes garnering significant research interest [7,9,12,13,14]. The dynamic reorganization of microtubule networks constitutes an essential determinant of neoplastic transformation, particularly in facilitating metastatic dissemination through cytoskeletal remodeling. Emerging evidence demonstrates that centrosomal microtubule hypernucleation promotes invasive potential in malignant cells, while non-centrosomal microtubule stabilization directly correlates with enhanced migratory capacity and pathological angiogenesis. Mechanistic studies have elucidated distinct oncogenic roles: (i) CAMSAP2 stabilizes non-centrosomal microtubules to facilitate hepatocellular carcinoma metastasis; (ii) γ-TuRC constitutive activation enhances centrosomal nucleation efficiency, potentiating invasive phenotypes; (iii) ASPM orchestrates oncogenic signaling via canonical Wnt/β-catenin pathway activation, driving solid tumor progression. Notably, the same family members may exhibit functional heterogeneity in different tumor types or molecular backgrounds. CAMSAP3 exhibits oncogenic properties in lung cancer, while CAMSAP1 is associated with immune escape in some tumors. This complexity suggests that the function of −TIPs may be finely regulated by the tumor microenvironment, epigenetic modifications and signaling pathway interactions.

While current research has elucidated fundamental aspects of −TIPs in oncogenesis, three critical translational challenges persist: (i) Mechanistic insights remain largely confined to preclinical models, with limited clinical validation in human tumor specimens; (ii) Context-dependent functional pleiotropy complicating precision oncology approaches; (iii) The mechanistic basis underlying post-translational modification-mediated regulation (phosphorylation, ubiquitination, acetylation) of microtubule affinity and neoplastic reprogramming remains elusive. In addition, the molecular mechanism by which cyclic RNA expression affects tumor progression adds a new research direction to this field.

In this paper, we systematically review the molecular features, functional networks and their roles in tumors of −TIPs such as CAMSAPs, γ-TuRC, and ASPM, focusing on their translational potentials in prognostic assessment and targeted therapies. By integrating multi-omics data and clinical evidence, this paper aims to provide a new perspective for resolving the association between microtubule dynamics and tumor malignant phenotypes, and to lay a theoretical foundation for the development of precise anti-tumor strategies based on microtubule minus-end regulation.

## 2. Classification and Role of −TIPs

The dynamic assembly and stability regulation of microtubules are crucial for cellular biological processes. While early investigations predominantly focused on microtubule plus-end-binding proteins, recent advances in molecular characterization techniques have revealed an expanding repertoire of minus-end-associated proteins offering critical insights for deciphering the microtubule minus-end regulatory network. As key regulators of microtubule minus-end stabilization, CAMSAPs modulate microtubule dynamics via distinct domains, orchestrating essential cellular processes including mitotic progression, synaptogenesis, and epithelial morphogenesis. The identification of evolutionarily conserved regulators including γ-TuRC, Asp/ASPM, and plant-specific Spiral2 has significantly broadened our understanding of minus-end regulatory mechanisms. Emerging evidence demonstrates that CEP170B coordinates with KIF2A to counterbalance CAMSAP2-mediated stabilization, elucidating sophisticated homeostatic control mechanisms governing microtubule minus-end dynamics.

The CAMSAPs play a pivotal role in microtubule minus-end regulation, with individual members employing distinct molecular mechanisms to modulate microtubule dynamics: CAMSAP1 exhibits dynamic tracking of microtubule minus-ends while maintaining structural integrity, whereas CAMSAP2/3 attenuate tubulin incorporation rates, thereby stabilizing microtubule integrity [5]. Recent paradigm shifts in microtubule research have established spatial organization regulation as a key research priority, particularly regarding microtubule crossover site. Notably, CAMSAP2/3 colocalize at these nodal points, where their depletion induces abnormal microtubule bundling that compromises intracellular trafficking efficiency [2]. In Drosophila, Patronin (the sole CAMSAP ortholog) was the first identified endogenous microtubule minus-end stabilizer, crucially coordinating presynaptic microtubule organization to maintain synaptic architecture [15], participating in epithelial morphogenesis through nucleation of non-centrosomal microtubule arrays [16], while also governing mitotic spindle scaling [6]. In Caenorhabditis elegans, PTRN-1 (the nematode CAMSAP homolog) demonstrates dual functionality in axonal regeneration and dendritic microtubule polarization [17], with emerging evidence implicating its novel role in actin cytoskeleton crosstalk for cytoskeletal homeostasis maintenance [18]. The distinct but coordinated functions, expression patterns, and pathological consequences of different CAMSAPs are systematically summarized in Table 1.

The γ-TuRC, recognized as a foundational microtubule minus-end binding complex, is structurally composed of γ-tubulin, GCP2-GCP6 proteins, actin, and regulatory subunits such as MZT1/MZT2. Microtubule assembly begins with the process of microtubule nucleation, γ-TuRC, as a major microtubule nucleation factor, is involved in microtubule nucleation both in vivo and in vitro [32]. However, Aher et al. proposed that γ-TuRC is an inefficient template for nucleation, which may be related to its extensive transition from the free state to the tightly bound microtubule state [1]. Notably, there is biochemical crosstalk between the microtubule minus-end regulatory pathways: Rai et al. proposed from in vitro reconstruction experiments that all CAMSAPs bind to the minus-end of γ-TuRC-connected microtubules, and that CAMSAPs drive the translocation of γ-TuRC from newly nucleated microtubules to generate free and stable microtubule minus-end [10].

Asp was one of the first mitotic genes identified in Drosophila, and the subsequent finding that mutations in its human homologue, ASPM, is the most common cause of autosomal recessive inheritance of primary microcephaly has attracted intense interest [12]. The Asp/ASPM-encoded proteins are localized at the spindle pole around the centrosome as well as at the outer portion of the central spindle, where they bind to the minus-end of microtubules that are separated from the centrosome [8]. Its subcellular localization is inextricably linked to its functional properties, and Asp/ASPM plays a central role in the regulation of cytokinesis, with depletion affecting processes such as stellate formation, spindle pole focusing, centrosome-spindle coupling, and spindle orientation [8].

The KAT8 regulatory complex subunit 3 gene (KANSL3) encoded protein was first identified by Meunier et al. as a −TIPs that autonomously binds, stabilizes, and promotes the assembly of microtubule minus-end, which are significantly enriched at the spindle poles during mitotic prophase. KANSL3 has been identified to play regulatory roles in multiple systems, and its depletion or deficiency leads to a variety of severe consequences: defects such as chromosomal misalignment, multipolar spindle formation and delayed midcycle division [9]; stochastic accumulation of nuclear anomalies in a pattern of genomic instability including chromosome fragmentation [33]; triggering a wide range of metabolic defects [34]; and triggering spectrum-specific defects in the inner cell mass of the mutant blastocyst as well as leading to early embryonic lethality [35].

Bolhuis et al. found in plant studies that Spiral2 is involved in the process of microtubule array reorientation in epidermal cells, and its role as a plant-specific −TIPs can stabilize newly formed microtubule arrays by autonomously accumulating at cortical microtubule minus-end and inhibiting microtubule contraction [36].

γ-TuRC is mainly involved in microtubule nucleation, and the discovery of proteins encoded by Asp/ASPM and KANSL3 extends the importance of negative regulation in the key position of cell division and genome stability maintenance, and the plant-specific protein Spiral2 stabilizes newly formed microtubules. Previous studies have found that CAMSAPs can effectively protect the microtubule minus-end from Kinesin-13-induced depolymerization in vitro, but microtubule minus-end depolymerization does occur in cells, suggesting that there is a substance that works synergistically with microtubule depolymerization enzymes to antagonize the effects of the CAMSAPs. Guan et al. discovered this phenomenon and explored it in depth, finding that CEP170B was able to bind to free microtubule minus-end and block minus-end growth, and that CEP170B in combination with KIF2A acted as a potent microtubule minus-end depolymerizing enzyme to antagonize the stabilizing effect of CAMSAPs on non-centrosomal microtubules [37]. This finding reveals an antagonistic mechanism to control the spatial distribution of microtubule minus-end and opens a new chapter in the study of −TIPs.

−TIPs regulate microtubule dynamics and spatial distribution through complex and sophisticated interaction mechanisms, thus maintaining cytoskeletal homeostasis and supporting a wide range of biological functions. The functions of the CAMSAPs are conserved and specific among species. Notably, the minus-end depolymerization mechanism synergistically mediated by CEP170B and KIF2A provides a new idea to understand the functional antagonism of CAMSAPs. These studies not only deepen the knowledge of the complexity of the microtubule minus-end regulatory network, but also provide potential targets for the resolution of disease mechanisms.

## 3. Characterization of the Domains of −TIPs

**Figure 2:**
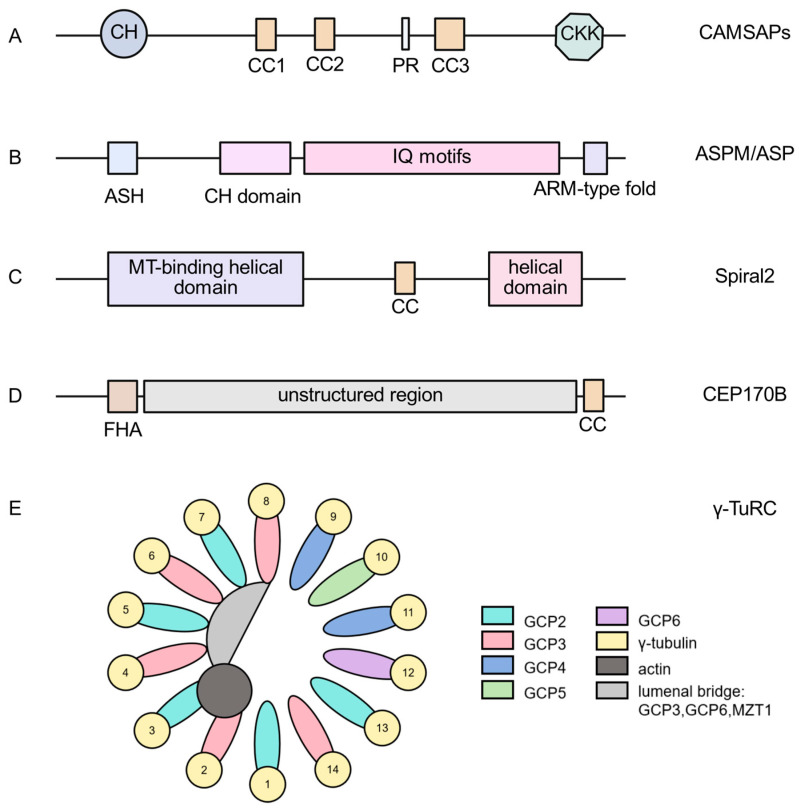
Domain organization of representative −TIPs. Schematic representations of the primary domain structures of key −TIPs. (**A**) CAMSAPs contain CH domains (blue circle), CC regions (pale yellow rectangle), proline-rich region (PR) (white rectangle), and the characteristic CKK domain (green octagon). (**B**) ASPM/ASP comprises an N-terminal region with Aspm-SPD 2-Hydin (ASH) domains (pale blue rectangle), a central region with multiple CH domains (purple red rectangle) and IQ motifs (pink rectangle), and a C-terminal region predicted to contain ARM repeats (purple rectangle). (**C**) Spiral2 consists of an N-terminal microtubule-binding helical domain (deep purple rectangle), a central CC region (pale yellow rectangle), and a C-terminal helical domain (pale pink rectangle). (**D**) CEP170B contains an N-terminal FHA domain (brown rectangle) and a C-terminal CC domain (pale yellow rectangle), connected by a long unstructured region (grey rectangle). (**E**) γ-TuRC is composed of 14 spokes, each containing γ-tubulin (yellow round) and a GCP protein (GCP2-GCP6) (ellipses of different colors), along with additional structural components including actin (dark gray round) and MZT (light gray semicircle). 1–14 is the number of spokes. Rectangles with different colors on the right side are an explanation of the components of the left figure.

The dynamic regulation of microtubule minus-end is essential for maintaining cell polarity, directional migration and material transport, a process that relies on a variety of −TIPs with specific domains. Different proteins are functionally differentiated through the combination of their unique domains, which precisely regulate the nucleation, stabilization and dynamic reorganization of the microtubule minus-end, forming a multilevel regulatory network. The domain organizations of major −TIPs discussed in this section are summarized in Figure 2.

CAMSAPs have several conserved domains, including the characteristic CKK domain, CH domain, CC domain, and proline-rich region, which collaborate with each other to jointly regulate microtubule behaviors. The CKK domain confers CAMSAPs with specific binding ability to the minus-end of microtubules through the mechanism of compression of the microtubule lattice and imposition of the right-handed superhelices modification [38]. Notably, the pioneering work by Meng et al. identified CAMSAP3 (also known as Nezha) as a key stabilizer of non-centrosomal microtubules, highlighting the fundamental role of its CKK domain [39]. Furthermore, structural studies have shown that the C-terminal region of CAMSAP3, including the α-helix, is critical for forming a stabilizing lattice at the minus end and enhancing microtubule affinity through dimerization [40]. Other domains play multiple roles in functional regulation: the CC domain is not only involved in regulating the extension direction of non-centrosomal microtubules, which plays a key role in controlling cell motility by coupling microtubules with actin filaments [41], but also necessary for Patronin binding to the minus-end of microtubules [5]. The critical importance of the C-terminal region, which houses the CKK domain, is powerfully demonstrated by in vivo studies. Research utilizing the Camsap3-dc mutant mouse model, which expresses a truncated protein lacking the C-terminal domain, has revealed severe phenotypes. These include a loss of apical-to-basal microtubule polarity in epithelial cells and defects in cellular organization, underscoring the indispensable role of an intact C-terminal domain for proper microtubule anchoring and function in tissue homeostasis [31]. The CH domain, as a common structural module of cytoskeletal regulatory proteins, may mediate the interaction between CAMSAPs and actin or microtubules [18]. Particularly in Patronin, the synergistic effect of CKK and CH domains has an important regulatory function on neuromuscular junction development [15]. This multi-domain synergistic mechanism enables the CAMSAPs to precisely regulate the spatiotemporal dynamic properties of microtubule networks.

Vertebrate γ-TuRC exists in an open left-handed helical conformation with a core backbone consisting of an arrangement of 14 spokes, each of which is formed by the binding of one GCP protein to one γ-tubulin via the GRIP2 domain of GCP. All GCP proteins contain conserved GRIP1 and GRIP2 domains that mediate interactions with γ-tubulin. Notably, unlike other GCP proteins, GCP6 contains an extended insertion domain that consists of nine repetitive sequences and a highly α-helicalized region. Through this special structure, GCP6 is able to specifically interact with the N-terminus of GCP2 and GCP5, thereby driving the precise assembly of the γ-TuRC three-dimensional structure. Further studies revealed that actin is also a structural component of γ-TuRC, which binds directly to γ-tubulin (spoke 2) through the D-loop [11]. This mode of interaction not only reveals the structural localization of actin in γ-TuRC, but also implies that it may be involved in the microtubule nucleation process by regulating the conformational changes in the complex.

The modular structure of Asp/ASPM proteins consists of three parts: the N-terminal, the central region, and the C-terminal, and each domain regulates its biological function through synergistic interactions. The N-terminal region contains two key domains: the Hydin domain, which is hypothesized to have microtubule-binding activity [42], and the spatially overlapping MSP domain, the function of which is not yet clear and may be involved in unknown molecular interactions [43]. The central region contains multiple CH domains, which are responsible for microtubule binding function, and many IQ repeats are also distributed in this region [43,44]. The C-terminal region may contain HEAT repeats [8], the specific function of which is unknown, but studies have shown that it may not be related to the microtubule minus-end binding function [43], suggesting that this region may achieve functional expansion by recruiting other regulatory factors or participating in protein complex assembly [42].

CEP170B contains an N-terminal FHA domain and a C-terminal convoluted helical domain connected by a long unstructured region. Current studies have shown that CEP170B does not bind to other proteins through its domain when interacting with them, but rather binds to the domains of other proteins through extended fragments located in the unstructured middle region. Its N-terminal region has minus-end specificity and is able to inhibit minus-end growth in a dose-dependent manner, and its C-terminal region provides additional microtubule lattice binding affinity and has the ability to track contractile ends [37].

Spiral2 consists of three regions: an N-terminal microtubule-binding helical domain, a convoluted helical region, and a C-terminal helical domain [45]. The N-terminal domain is responsible for microtubule binding, the convoluted helical region is used for oligomerization, and the C-terminal helical domain is highly α-helical, but the functional significance remains to be elucidated [45,46]. In animals, CAMSAPs have been identified as a functional homolog of Spiral2. Unlike Spiral2, the CKK domain of CAMSAPs belongs to the α + β domains [46,47], which may also explain their different functions; while CAMSAP2/3 promote microtubule assembly at the minus-end, Spiral2 and Patronin only inhibit microtubule disassembly and do not enhance microtubule polymerization [6,47,48,49].

Different −TIPs achieve functional differentiation through the combination of specific domains: for example, the CAMSAPs rely on the CKK domain to bind to the microtubule minus-end, γ-TuRC uses the extended domain of GCP6 to drive the molecular assembly, and Asp/ASPM anchors microtubules through the CH domain. The diverse combinations of these domains together construct a precise regulatory system of microtubule minus-end, providing a molecular basis for the establishment of cell polarity and movement regulation.

## 4. Relationship Between −TIPs and Tumor Prognosis

−TIPs play an important role in maintaining microtubule function and dynamic homeostasis, and their aberrant expression may be associated with tumor development, prognosis, and chemotherapy resistance. Currently, more studies on the relationship between −TIPs and tumor prognosis have been conducted on CAMSAPs and ASPM, while fewer studies have been conducted on the relationship between γ-TuRC, KANSL3 and CEP170B and tumor prognosis, mainly focusing on gliomas [3,50,51].

CAMSAPs, as key regulators of microtubule dynamics, exhibit multifaceted roles in tumorigenesis and development. The functions of this family members in tumors show significant heterogeneity, with CAMSAP1 and CAMSAP2 mainly exerting pro-tumorigenic effects, while CAMSAP3 exhibits tumor suppressor properties.

CAMSAP1 shows different roles in different tumors: CAMSAP1 mutation can be used as a platinum-based chemotherapeutic drug sensitivity marker in small cell lung cancer [21]; its high expression has been found to correlate with advanced progression and poor prognosis in hepatocellular carcinoma by bioinformatics analysis [4]. Notably, cyclic RNA circCAMSAP1 is commonly highly expressed in colorectal, hepatocellular and nasopharyngeal cancers and plays a key role by promoting tumor cell proliferation, migration or invasion [52,53,54]. In hematologic tumors, CAMSAP1 expression levels are valuable for prognostic assessment of acute lymphoblastic leukemia [55].

The oncogenic mechanism of CAMSAP2 mainly involves several classical signaling pathways: in hepatocellular carcinoma, inhibition of HDAC6 through the JNK/c-Jun pathway promotes microtubule acetylation-mediated tumor metastasis [7]; in colorectal cancer, activation of the JNK/c-jun/MMP-1 signaling axis drives cellular invasion [13]; and in gastric cancer, it promotes epithelial–mesenchymal transition (EMT) progression through upregulation of TGF-β signaling [56]. The pro-metastatic role of this protein is particularly prominent in non-small cell lung cancer, both reversing the oncogenic effect of miR-2355-5p and promoting metastasis by regulating RASAL2 degradation [14,57]. Notably, its involvement in the mechanism of HBV-mediated hepatocarcinogenesis provides a new perspective for viral carcinogenesis research [58].

Unlike other members of the family, CAMSAP3 has tumor suppressor potential: it inhibits cell migration through an EMT-dependent mechanism [59]; and it acts through multiple mechanisms in lung cancer, inhibiting both tumor invasion and angiogenesis by negatively regulating HIF-1α mRNA stability [60]; and inducing a cellular senescence-like phenotype by mediating cell cycle arrest through ERK phosphorylation regulation [27]. Recent studies revealed its critical position in autophagic cell death, with HDAC inhibitors inducing tumor cell death by enhancing CAMSAP3 interactions with acetylated HMGB1 [61]. Moreover, its prognostic value in endometrial cancer suggests its broad-spectrum oncogenic potential [62].

ASPM, as an evolutionarily conserved oncogene, plays a central role in a variety of solid tumors through the Wnt/β-catenin signaling axis: it drives the EMT process through β-catenin nuclear translocation in colorectal cancer [63]; and regulates the cell cycle and Wnt signaling in glioblastoma [64]. Its pro-carcinogenic mechanisms are highly diverse: it regulates cell growth through CDK4 in lung squamous carcinoma [65]; regulates cell motility by stabilizing KIF11 in interstitial thyroid carcinoma [66]; and forms a pro-carcinogenic axis with METTL3 in hepatocellular carcinoma [67]. Notably, ASPM expression levels are closely associated with the development of digestive system tumors such as cholangio carcinoma, esophageal cancer, and pancreatic ductal adenocarcinoma [68,69,70], and its potential as a cross-cancer prognostic marker has been validated in multi-tumor species such as bladder cancer and renal cancer [71,72,73,74]. In addition, ASPM isoform 1 (ASPM-i1) is associated with poor prognosis in patients with small cell lung cancer or gastric cancer and is critical for tumorigenesis in these malignancies [75,76].

−TIPs play an important role in regulating tumor cell biology. CAMSAPs have different functions in different types of tumors (see Table 2), among which CAMSAP1 and CAMSAP2 exhibit pro-oncogenic roles in a variety of solid tumors, while CAMSAP3 has an oncogenic role in some tumors. ASPM is closely associated with poor prognosis of a variety of cancers and can promote tumor progression through signaling pathways such as Wnt/β-catenin. These results suggest that multiple −TIPs have potential value in tumor diagnosis, prognosis and treatment.

## 5. Mechanisms of −TIPs in Cancer Promotion

The dynamic balance of the microtubule system is a central element in maintaining cellular homeostasis, and its dysfunction can lead to abnormal cell division and tumorigenesis. As a key regulator of microtubule minus-end, the pro-tumorigenic mechanism of −TIPs is still unclear, which may involve cell cycle regulation, cytoskeleton alteration, angiogenesis and other aspects.

−TIPs may affect cell proliferation and apoptosis by regulating cell cycle changes. For example, upregulation of circCAMSAP1 induces G2/M phase arrest in nasopharyngeal carcinoma cells [54], and knockdown of circCAMSAP1 induces G1 phase cell cycle arrest in colorectal carcinoma [52]; CAMSAP3 deletion inhibits the expression level of the cell cycle protein D1 and induces G1 cell cycle arrest, which leads to irreversible growth arrest and tumor aggressiveness [27]. This spatiotemporal specificity of cell cycle regulation suggests that −TIPs may dynamically regulate the proliferative state of tumor cells through multiple mechanisms.

The core of tumor cell metastasis lies in the ability to establish cell polarity and directional migration. Studies have shown that CAMSAP1 dysfunction can lead to abnormal neuronal polarity and impaired migration [77], a mechanism that may be hijacked in tumor metastasis: −TIPs remodel the spatial conformation of the cytoskeletal network by modulating microtubule dynamic stability. Its aberrant expression can disrupt the mechanical balance between the anterior and posterior cell poles, contributing to the acquisition of an invasive phenotype by tumor cells. Notably, −TIPs can construct a metastasis-friendly cytoskeletal microenvironment by coordinating the interaction of microtubules with other cytoskeletal protein networks, such as actin [78].

Tumor angiogenesis is dependent on the directional migration capacity of endothelial cells. CAMSAP2 has been found to play a key role in vascular sprout formation by stabilizing non-centrosomal microtubules: it maintains the polarized distribution of microtubules in single-cell protrusions and ensures the continuous migration required for angiogenesis [79]. This unique microtubule stabilization mechanism provides an adequate nutrient supply to the tumor and establishes a “high speed pathway” for metastasis.

Overall, the relationship between −TIPs and tumor prognosis is complex, and the specific role may depend on factors such as tumor type and expression level of −TIPs. Therefore, more studies are needed to delve into the mechanism of −TIPs in different tumor types, as well as their potential applications in tumor prognostic assessment and treatment.

## 6. Translational Studies of −TIPs

With the increasing morbidity and mortality of malignant tumors in China and the emergence of various novel anti-cancer drugs, molecular targeted therapy has become an important direction in tumor treatment. Microtubules, as key structures for cell division, migration and material transportation, can be significantly affected by kinetic disorders, and thus microtubules have become a core target for cancer therapy. Microtubule-targeting agents that directly bind to microtubules are currently the most common and effective chemotherapeutic agents, for example, various anti-microtubule drugs such as paclitaxel and alkaloids have been widely used to enhance the treatment of malignant tumors, such as lung and pancreatic cancers.

However, due to the complexity of microtubule regulation, there are problems of resistance and drug toxicity of such drugs, which limit the effectiveness of their clinical application. Therefore, exploring new therapeutic strategies for microtubule inhibition has become the focus of current research. Studies have shown that abnormal enhancement of centrosomal microtubule nucleation ability promotes tumor cell invasiveness, whereas blocking γ-TuRC function effectively reverses the high nucleation ability of centrosomes, which may impede the invasive and metastatic behaviors of tumor cells with redundant centrosomes, and thus targeting γ-TuRC may provide a viable alternative to perturbing tumor cells [51]. In addition, −TIPs may also play a key role in tumorigenesis and development by influencing the post-translational modification process of non-centrosomal microtubules. For example, CAMSAP2 promotes hepatocellular carcinoma metastasis by promoting non-centrosomal microtubule acetylation [7]. Therefore, future studies should further resolve the specific mechanisms of −TIPs in different tumor types, with the aim of providing more effective solutions for precision tumor therapy.

## 7. Discussion

−TIPs, as key factors regulating microtubule dynamics and stability, are closely related to tumorigenesis and development in terms of their expression and functional abnormalities. In this paper, we systematically reviewed the molecular features, functional mechanisms and their potential roles in tumors of −TIPs, such as CAMSAPs, γ-TuRC, ASPM, and KANSL3-encoded proteins. It was shown that these proteins affect core biological processes such as cell division, polarity establishment, material transport and cell migration by regulating the dynamic assembly and disassembly of microtubule minus-end. For example, CAMSAP2 is involved in tumor cell invasion and metastasis by stabilizing the minus-end of non-centrosomal microtubules; abnormal activation of γ-TuRC, a core factor in microtubule nucleation, may promote the over-assembly of centrosomal microtubules, thereby enhancing the malignant phenotype of tumor cells; and ASPM may be involved in tumor progression by regulating spindle microtubule dynamics. Notably, the role of −TIPs in tumors is significantly heterogeneous. The same family members may exhibit different biological effects in different tumor types or molecular contexts. For example, CAMSAP1 promotes tumor progression in hepatocellular carcinoma, but its mutation correlates with elevated chemosensitivity in small cell lung cancer; CAMSAP2 significantly promotes invasive metastasis in a variety of solid tumors such as hepatocellular carcinoma and colorectal carcinoma; and CAMSAP3 can negatively regulate cell migration through an EMT-dependent mechanism. This complexity suggests that the function of −TIPs may depend on the tumor microenvironment, epigenetic modifications, and interactions with other signaling pathways. In addition, antagonistic mechanisms between −TIPs reveal the importance of the fine balance of the microtubule dynamics regulatory network to maintain cellular homeostasis, and its imbalance may become a critical node for tumor cells to escape from therapy. At the translational medicine level, intervention strategies targeting −TIPs show potential therapeutic promise. For example, inhibition of γ-TuRC function reverses the centrosomal hypernucleation ability of tumor cells, reduces cell invasiveness and metastatic potential, and provides a new direction in the search for tumor therapeutic targets. However, existing studies are mostly limited to cell and animal models, and clinical translation still faces challenges.

Furthermore, although this review focuses on the roles of −TIPs in tumorigenesis and metastasis, it is noteworthy that CAMSAPs also play indispensable roles in various non-cancerous tissues and developmental processes. Evidence from CAMSAP-deficient mouse models demonstrates significant microtubule-related phenotypes across multiple organs, including loss of polarity in intestinal epithelial cells [31], structural abnormalities in sperm flagella [20], impaired tracheal ciliary function [30], disrupted renal tubule morphogenesis [28], and neuronal migration defects [29]. These phenotypes collectively indicate conserved functions for CAMSAPs in fundamental processes including cell polarity maintenance, ciliogenesis, cytoskeletal stability, and directed cell migration. These same processes, in particular the establishment of cell polarity and migratory capacity, are key for tumor cells to acquire invasive and metastatic properties. Consequently, elucidating the physiological functions of CAMSAPs in normal tissues is crucial for understanding the pathology of their dysregulation in cancer and provides critical insights into how microtubule stability dysfunction contributes to various diseases. Future studies that integrate the roles of CAMSAPs in development, tissue homeostasis, and cancer will be essential for a comprehensive evaluation of −TIPs as therapeutic targets.

Although this review has focused on the functions of CAMSAPs, γ-TuRC, and ASPM in animal cells and cancer, it is important to recognize that the principles of microtubule minus-end regulation are well-documented in the plant kingdom. For instance, in Arabidopsis thaliana, the Spiral2 protein family, which are functional analogues of CAMSAPs, sustains persistent microtubule growth by suppressing depolymerization [45]. Although plant systems are not directly associated with human cancer pathogenesis, the core regulatory mechanism of the Spiral2 family, which stabilizes microtubule minus-ends to establish and maintain specific polarized cellular structures, is functionally analogous to that of CAMSAPs in cancer cell migration, polarized growth, and angiogenesis. This cross-kingdom conservation suggests that the regulation of microtubule minus-end stability represents an ancient and fundamental mechanism underlying the construction of complex cellular architectures and directed cell movement. Therefore, investigating plant-specific −TIPs such as Spiral2 will not only deepen our evolutionary understanding of microtubule biology but could also inspire novel intervention strategies for microtubule-related diseases, including cancer invasion and metastasis.

In conclusion, the study of −TIPs, as an important bridge connecting cytoskeleton regulation and tumor malignant phenotypes, has not only deepened the understanding of microtubule biological mechanisms but also provided new molecular targets for tumor-targeted therapies. With the advancement of technology and the deepening of interdisciplinary cooperation, translational research in this field is expected to bring breakthroughs in tumor treatment strategies.

## Figures and Tables

**Table 1:** Functional Characterization of CAMSAPs.

Member	Primary Function	Expression Localization	Phenotypes Upon KO/Knockdown/Mutation	Associated Diseases/Phenotypes
CAMSAP1	Dynamically tracks microtubule minus-ends and maintains structural integrity.	Neurons [19], spermatozoa [20], lung [21], liver [22], vascular smooth muscle cells et al. [23]	Formation of multiple axon-bearing neurons; deformed sperm nuclei and flagella; activation of anti-tumor immunity; inhibition of hepatic stellate cell activation; enhanced proliferation, migration, and phenotypic switching of vascular smooth muscle cells.	Cortical development malformations; oligoasthenozoospermia; lung cancer; liver fibrosis; in-stent restenosis.
CAMSAP2	Co-decorates and stabilizes growing microtubule minus-ends alongside CAMSAP3.	Pancreatic β-cells [24], liver [7], lung [14], mitral valve cells [25], neurons et al. [26]	Impaired glucose-stimulated insulin secretion; transition of non-centrosomal microtubule arrays to a centrosomal pattern; suppression of lung cancer cell motility in vitro and metastasis in vivo; impaired classic morphology of mitral valve cells; increased neurite outgrowth.	Diabetes; hepatocellular carcinoma; lung cancer; olfactory defects; epilepsy.
CAMSAP3	Co-decorates and stabilizes growing microtubule minus-ends alongside CAMSAP2.	Lung [27], kidney [28], neurons [29], trachea [30], intestine et al. [31]	Promotion of cellular senescence in lung cancer; cyst formation in kidneys; aberrant narrowing or fusion of the boundary between the striatum and septum; failure of multi-cilia to undergo synchronous beating; impaired positioning of nuclei and Golgi apparatus, and mitochondrial shaping.	Lung cancer; proximal renal tubule cyst formation; abnormally narrow lateral ventricles; tracheal ciliary dyskinesia; organ growth retardation and impaired physiological function.

**Table 2:** Relationship between −TIPs and tumor prognosis.

Molecular Name	Cancer Type	OS	Invasion (↑/↓)	Migration/ Metastasis (↑/↓)	Proliferation (↑/↓)	Key Findings and Mechanisms	Experimental System
Mutated CAMSAP1 [21]	SCLC	Long	—	—	—	Mutant activates anti-tumor immunity, mediates tumor cell apoptosis, and inhibits EMT.	Patient Sample Analysis
CAMSAP1 [4]	LIHC	Short	—	—	—	The AC145207.5/LINC01748-miR-101-3p-CAMSAP1 axis promotes tumor progression.	Bioinformatics Analysis
circCAMSAP1 [52]	CRC	Short	—	—	↑	Acts as a molecular sponge for miR-328-5p, relieving its repression of the transcription factor E2F1, thereby driving cell cycle progression and proliferation.	Patient Samples, In Vitro, In Vivo
circCAMSAP1 [53]	HCC	—	↑	↑	↑	The circCAMSAP1/miR-1294/GRAMD1A axis promotes oncogenic phenotypes.	Patient Samples, In Vitro, In Vivo
circCAMSAP1 [54]	NPC	—	↑	↑	↑	Forms a positive feedback loop with SERPINH1 and c-Myc to sustain tumor growth and invasion.	Patient Samples, In Vitro, In Vivo
CAMSAP2 [7]	HCC	Short	↑	↑	—	Suppresses HDAC6 via the Rac1/JNK/c-Jun pathway, promoting non-centrosomal microtubule acetylation, which enhances stability and facilitates metastasis.	Patient Samples, In Vitro, In Vivo
CAMSAP2 [13]	CRC	Short	↑	↑	→	Activates the JNK/c-Jun signaling axis, upregulating Matrix Metalloproteinase-1 (MMP-1) expression to promote extracellular matrix degradation and invasion.	Patient Samples, In Vitro, In Vivo
CAMSAP2 [56]	GC	Short	↑	↑	—	Promotes EMT potentially via upregulation of the TGF-β signaling pathway.	Patient Samples, In Vitro, In Vivo
CAMSAP2 [57]	NSCLC	—	↑	↑	↑	The CircSOD2/miR-2355-5p/CAMSAP2 axis contributes to malignant behaviors.	Patient Samples, In Vitro
CAMSAP2 [14]	NSCLC	Short	—	↑	→	Promotes RASAL2 degradation, leading to subsequent activation of the ERK signaling pathway and enhanced cell migration.	Patient Samples, In Vitro, In Vivo
CAMSAP3 [60]	LUAD	Long	↓	↓	—	Binds NCL to negatively regulate HIF-1α mRNA stability, thereby inhibiting tumor angiogenesis and invasion.	Patient Samples, In Vitro, In Vivo
CAMSAP3 [27]	NSCLC	—	—	↓	↑	Depletion inhibits ERK phosphorylation and Cyclin D1 expression, inducing G1 cell cycle arrest and senescence-like phenotypes.	In Vitro, In Vivo
CAMSAP3 [61]	LC	Long	—	—	—	Interacts with acetylated HMGB1 to enhance autophagic cell death upon TSA treatment.	Patient Samples, In Vitro
ASPM-i1 [75]	SCLC	Short	↑	—	—	Stabilizes key Hedgehog signaling components (GLI1, DVL3, SMO), enhancing cancer stemness and progression.	Patient Samples, In Vitro, In Vivo
ASPM-i1 [76]	GC	Short	↑	—	—	Cooperates with FOXM1 and β-catenin to potentiate Wnt/β-catenin signaling.	Patient Samples, In Vitro, In Vivo
ASPM [63]	CRC	Short	↑	↑	→	Promotes β-catenin nuclear translocation, activating Wnt/β-catenin signaling to drive EMT, migration, and invasion.	Patient Samples, In Vitro, In Vivo
ASPM [65]	LSCC	Short	—	↑	—	Contributes to progression potentially by regulating CDK4 and cell cycle progression.	Patient Samples, In Vitro
ASPM [72]	PRCC	Short	↑	↑	↑	Promotes malignant phenotypes, partially through activation of the Wnt/β-catenin signaling pathway.	Patient Samples, In Vitro, In Vivo
ASPM [66]	ATC	—	↑	↑	—	Binds to and stabilizes kinesin KIF11 by inhibiting its ubiquitin-mediated degradation, thereby promoting cell motility.	Patient Samples, In Vitro, In Vivo
ASPM [67]	LIHC	Short	↑	↑	↑	The METTL3-mediated m6A methylation enhances ASPM mRNA stability and expression, forming a pro-oncogenic axis.	Patient Samples, In Vitro, In Vivo

“↑”: Promotion; “↓”: Inhibition; “→”: No Effect; “—“: Not Reported.

## Data Availability

No new data were created or analyzed in this study. Data sharing is not applicable to this article.

## Abbreviations

The following abbreviations are used in this manuscript:

CAMSAPs	calmodulin-regulated spectrin-associated proteins
γ-TuRC	γ-tubulin ring complex
ASPM	abnormal spindle microtubule assembly
KANSL3	KAT8 regulatory complex subunit 3 gene
SCLC	small-cell lung cancer
LIHC	liver hepatocellular carcinoma
CRC	colorectal cancer
NPC	nasopharyngeal carcinoma
HCC	hepatocellular carcinoma
NSCLC	non-small-cell lung cancer
LC	lung cancer
LUAD	lung adenocarcinoma
LSCC	Lung squamous cell carcinoma
GC	gastric cancer
PRCC	papillary renal cell carcinoma
ASPM-i1	ASPM isoform 1
ATC	anaplastic thyroid carcinoma
EMT	epithelial–mesenchymal transition
GRAMD1A	GRAM Domain Containing 1A
JNK	c-Jun N-terminal kinase
MMP-1	Matrix Metalloproteinase-1
CircSOD2	circRNA SOD2
TGF-β	transforming growth factor-β
RASAL2	Ras protein activator-like 2
ERK	extracellular signal-regulated kinase
Rac1	Ras-related C3 botulinum toxin substrate 1
HDAC6	histone deacetylases 6
HMGB1	high mobility group box 1
TSA	trichostatin A
NCL	nucleolin
HIF-1α	hypoxia-inducible factor-1α
CDK4	cyclin dependent kinases 4
GLI1	glioma-associated oncogene homolog 1
FOXM1	Forkhead box protein M1
Dvl-3	disheveled-3
Smo	Smoothened
KIF11	kinesin family member 11
METTL3	methyltransferase-like 3
m6A	N6-methyladenosine
